# Supplementary material for: C. elegans enteric motor neurons fire synchronized action potentials underlying the defecation motor program
Source: Nat Commun. 2022 May 19;13:2783. doi: 10.1038/s41467-022-30452-y (PMC9120479; doi:10.1038/s41467-022-30452-y)
Supplement: Supplementary file 1 — Supplementary Information [file 41467_2022_30452_MOESM1_ESM.pdf]

C. elegans enteric motor neurons fire synchronized action potentials underlying the defecation motor program

Jingyuan Jiang, Yifan Su, Ruilin Zhang, Haiwen Li, Louis Tao, Qiang Liu

Supplementary Information

Supplementary Figures 1-6

Supplementary Method

Supplementary Table 1

Supplementary Movies 1-2

Supplementary Reference

Supplementary Figure 1.

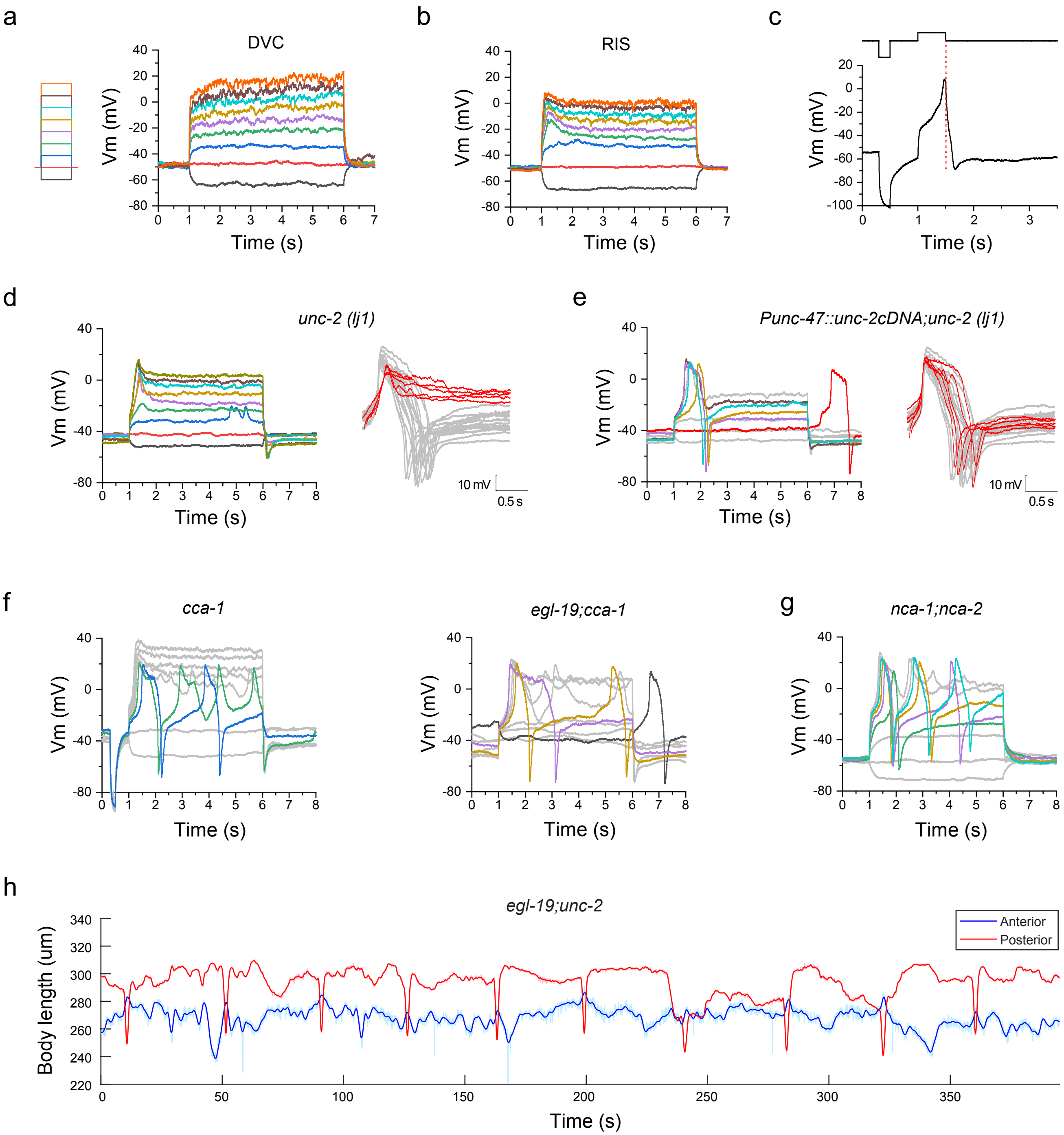

Supplementary Figure 1. AVL action potential is mediated by UNC-2
a - b Representative current-clamp recording traces from DVC and RIS in wild-type animals. **c** Wild-type AVL under a 0.5 s stimulation. The upstroke of an action potential was truncated by the early onset of negative spike. **d** Representative current-clamp recording traces from AVL in a second *unc-2* loss-of-function allele *lj1.* Left: Representative recording traces under a series of current injections steps. Right: overlay of recording traces from *unc-2(lj1)* (red) aligned to wild-type action potential spikes (same as in Fig. 2a but in gray). **e** Representative current-clamp recording traces from AVL in *unc-2(lj1)* rescued animals. **f - g** Representative action potential traces recorded from AVL in *cca-1, egl-19;cca-1* and *nca-1;nca-2* mutants. **h** Representative body-muscle contractions recorded from freely-moving animals in *egl-19;unc-2* double mutant. The behavior tracking method was the same as for Fig. 5-7 (See Method section). The traces were smoothed version of the curves in lighter color (Smoothing used local regression to a second order polynomial). Source data are provided as a Source Data file.

Supplementary Figure 2.

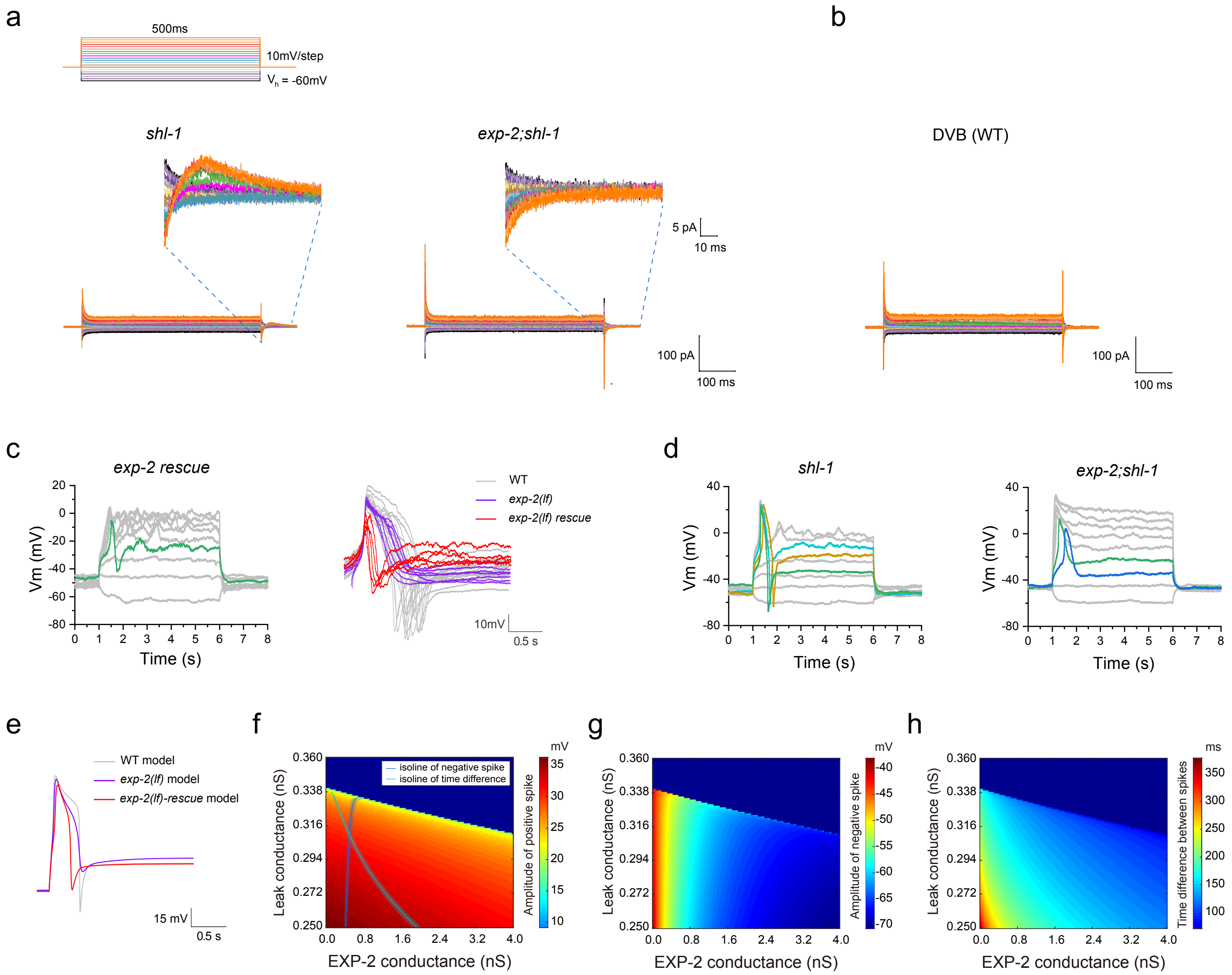

Supplementary Figure 2. AVL negative spike is mediated by EXP-2

a - b Representative voltage-clamp traces recorded from AVL in *shl-1, exp-2;shl-1* mutant animals (**a**) and DVB in wild-type animals (**b**). **c** Representative action potential traces recorded from AVL in *exp-2* rescued animals. Right: overlay of recording traces from *exp-2* rescued (red) aligned to wild-type and *exp-2 (lf)* action potential spikes (same as in Fig. 3d). **d** Representative action potential traces recorded from AVL in *shl-1* and *exp-2;shl-1* mutants. *shl-1* AVL fires WT-like action potentials. *exp-2;shl-1* AVL fires *exp-2(lf)*-like action potentials. **e** Representative action potential simulations for WT, *exp-2(lf)* and *exp-2(lf)-rescued* AVL. Based on the parameters of *exp-2(lf)* model, the maximum EXP-2 conductance (g_{EXP2}) and the leak conductance (g_L) was set to 0.5 nS and 0.31 nS, respectively, to model the *exp-2(lf)-rescued* AVL. **f - h** Characterization of the action potential in parameter space. The positive (**f**) and negative (**g**) spike amplitude, and the time difference between the positive and negative spikes (**h**) as functions of g_{EXP2} and g_L. Dark blue regions denote parameter values for which the model is non-excitable. The isolines of the negative spike amplitude and the time difference between the positive and negative spikes were plotted on (**f**). Intersection of the isolines at (0.5, 0.31) indicates the *exp-2(lf)-rescued* AVL action potential plotted in panel 1. (The shading denotes the range for modeling tolerance.) Source data are provided as a Source Data file.

Supplementary Figure 3.

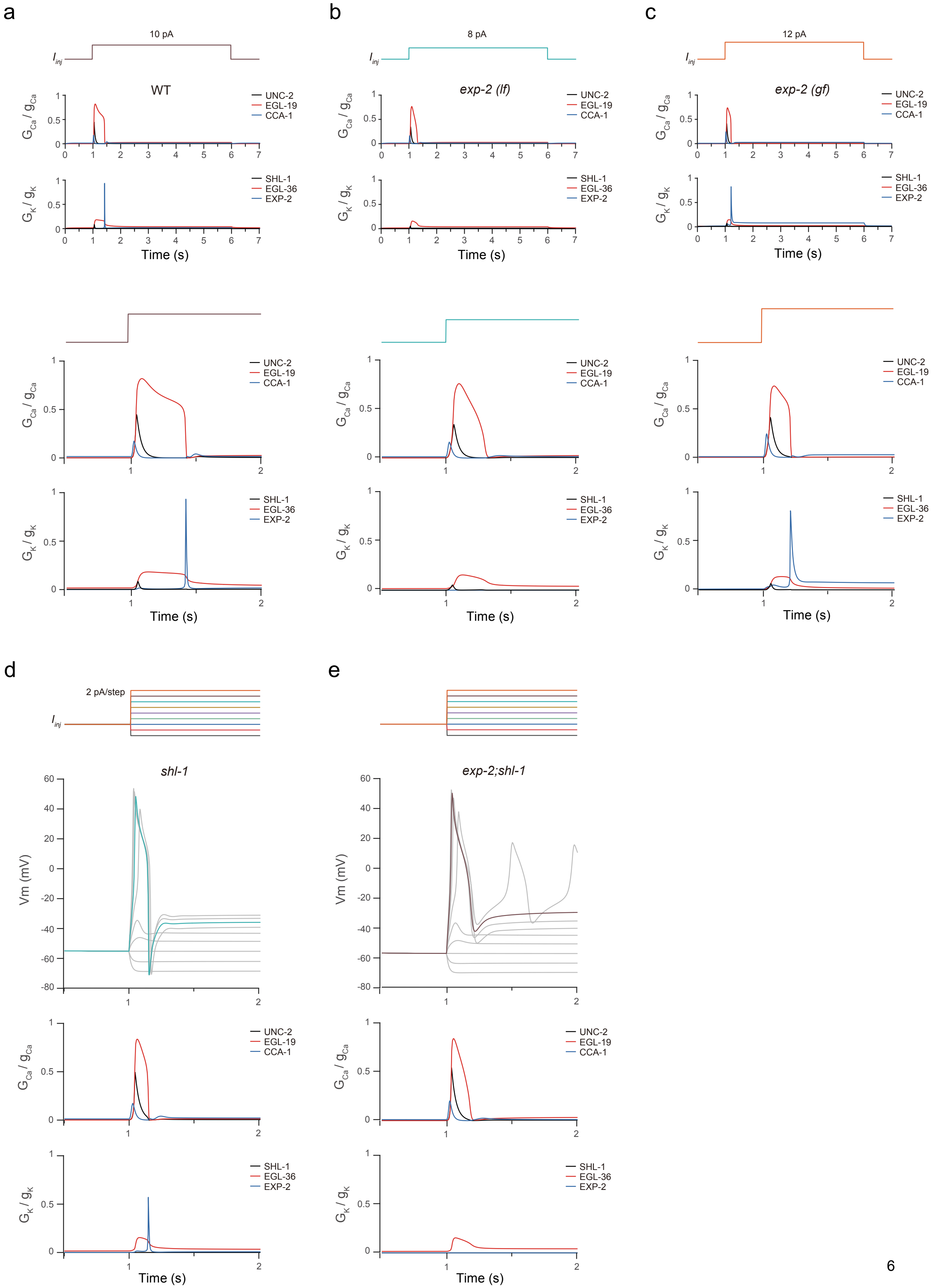

Supplementary Figure 3. Modeling of AVL action potentials

a - c Temporal evolution of the normalized conductance of each modeled calcium current and potassium current in wild-type, *exp-2(lf)* and *exp-2(gf)* AVL. Top: current-injection stimulation used. The stimulating currents are set to 10 pA, 8 pA and 12 pA for wild-type, *exp-2(lf)* and *exp-2(gf)* AVL, respectively. The normalized conductances correspond to the colored traces of the current clamp simulations of Fig. 4c, d, and e in the main text. The bottom two panels are the zoomed-in view of the panels above. **d - e** Representative action potential simulations and the normalized conductance of each modeled current for *shl-1* mutant and *exp-2;shl-1* double mutant AVL. The temporal evolution of the normalized conductance is color matched with the corresponding current clamp simulation.

Supplementary Figure 4.

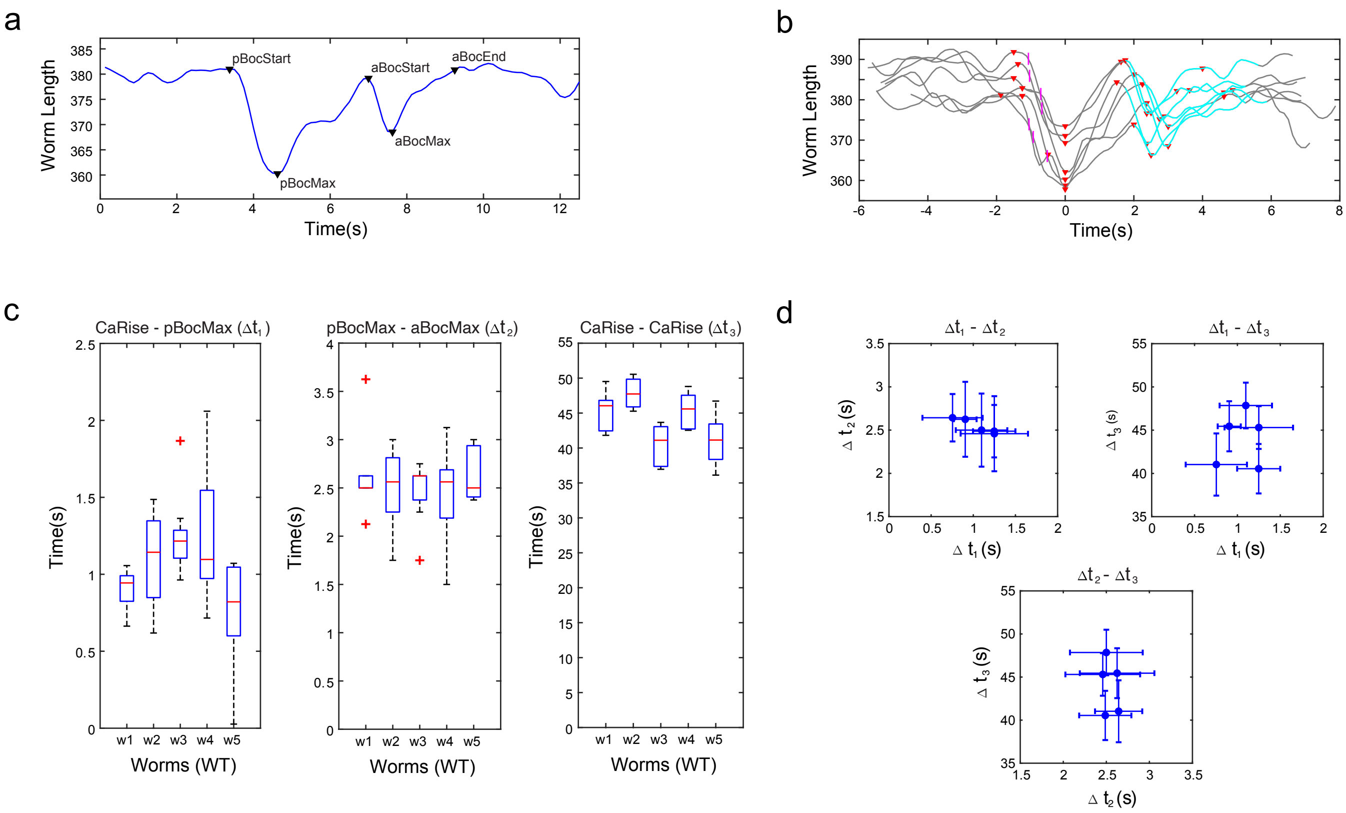

Supplementary Figure 4. Behavior tracking of defecation motor steps
a Definition of DMP events (start of pBoc, peak of pBoc, start of aBoc, peak of aBoc, end of aBoc) based on the change of worm body length. **b** Changes of body length were aligned to the peak of aBoc in several cycles. Pink: average timing when intestinal calcium activities reach half-peak in each segment. Blue: when AVL is active estimated by visual inspection. **c** Statistical comparison of DMP event intervals between wild-type individuals. Each column represents a wild-type individual. Data are presented in box plot. Box center: median value; box top: third quartile; box bottom: first quartile; The whiskers extend to the most extreme data points not considered outliers, and the outliers are plotted individually using the '+' symbol. (worm 1: n=8; worm 2: n=8; worm 3: n=10; worm 4: n=12; worm 5: n=8.). CaRise was defined as the average timing when intestinal calcium activities reach half-peak in each segment. As imaging processing is difficult when the worm is in a coiled shape, the cycles analyzed in an individual here were not all consecutive, and CaRise-CaRise was only calculated between consecutive cycles. **d** Correlation between the intervals examined in C (Δt_1: CaRise-pBocMax, Δt_2: pBocMax-aBocMax, Δt_3: CaRise-CaRise). Data are presented as mean values +/- SD. Each point represents the mean value of a wild-type individual and the error bar is SD (worm 1: n=8; worm 2: n=8; worm 3: n=10; worm 4: n=12; worm 5: n=8). Source data are provided as a Source Data file.

Supplementary Figure 5.

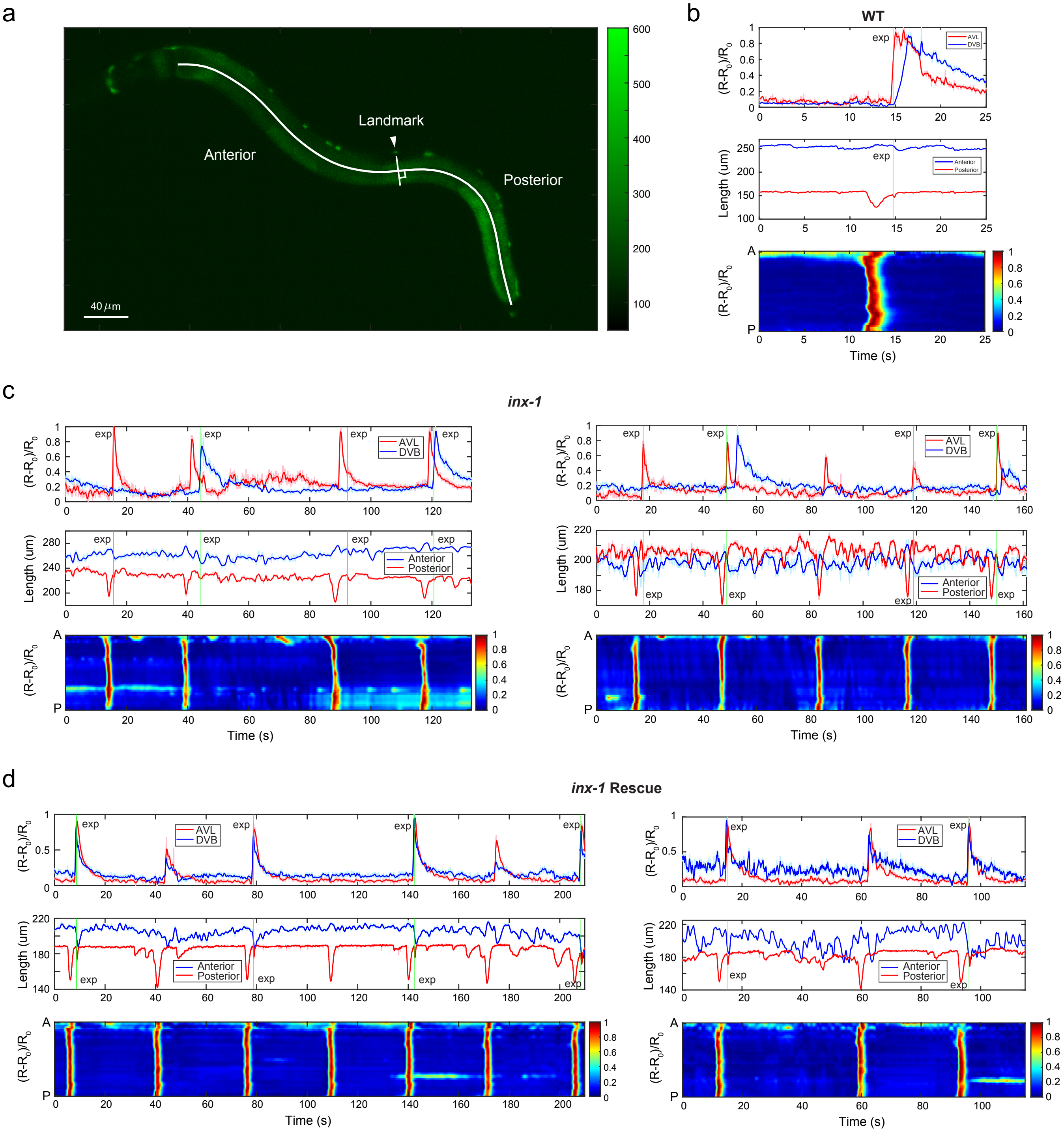

Supplementary Figure 5. *inx-1* mutant results in desynchronized firing of AVL and DVB

a Worm centerline was divided into anterior and posterior half, using VD/DD motor neurons as landmarks. All duo-color calcium imaging experiments performed (n=69) went through this analysis. **b - d** Representative simultaneous behavioral tracking and calcium imaging recording traces in wild-type (**b**), *inx-1* (**c**), and *inx-1* rescued (**d**) animals. Top: normalized calcium activities of AVL (red) and DVB soma (blue). Middle: Representative trace showing the length change of the anterior (red) and posterior (blue) half of the centerline. The traces were smoothed version of the curves in lighter color (Smoothing used local regression to a second order polynomial). Bottom: Representative normalized intestinal calcium activities along the worm body axis (A: Anterior, P: Posterior). Vertical lines: Timing of expulsion. Source data are provided as a Source Data file.

Supplementary Figure 6.

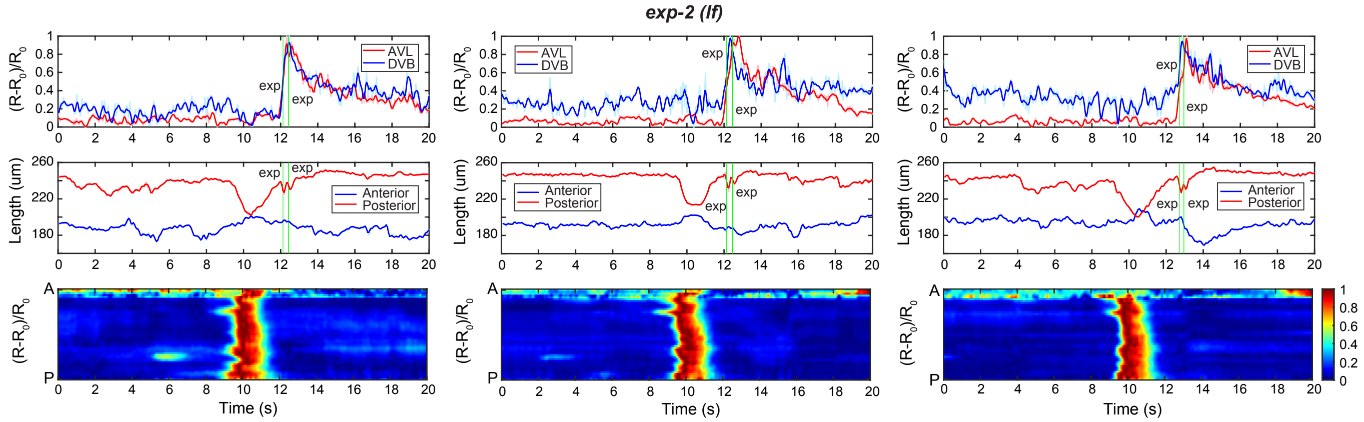

Supplementary Figure 6. Secondary expulsion events within a defecation cycle in *exp-2 (lf)* mutant

Three representative simultaneous behavioral tracking and calcium imaging recording traces in *exp-2(lf).* Top: normalized calcium activities of AVL (red) and DVB soma (blue). Middle: Representative trace showing the length change of the anterior (red) and posterior (blue) half of the centerline. The traces were smoothed version of the curves in lighter color. (Smoothing used local regression to a second order polynomial). Bottom: Representative normalized intestinal calcium activities along the worm body axis (A: Anterior, P: Posterior). Vertical lines: Timing of expulsion. Source data are provided as a Source Data file.

Supplementary Methods

Modeling of AVL action potential

To model the dynamics of the AVL membrane potential, we adopted a Hodgkin-Huxley formulation by modeling the ion channels that are participating in AVL action potentials. From our experiments and the open access databases Wormbase and CeNGEN, we chose six voltage-gated calcium and potassium channels (UNC-2, EGL-19, CCA-1, SHL-1, EGL-36 and EXP-2) and NCA sodium leak channels to constitute the AVL model. Therefore, the dynamics of the membrane potential of AVL is described by

$$C\frac{\mathrm{d}V}{\mathrm{d}t} = -\left(I_{\mathrm{UNC2}} + I_{\mathrm{EGL19}} + I_{\mathrm{CCA1}} + I_{\mathrm{SHL1}} + I_{\mathrm{EGL36}} + I_{\mathrm{EXP2}} + I_{\mathrm{NCA}} + I_{\mathrm{L}}\right) + I_{inj} \quad (1)$$

where C is the membrane capacitance. In addition to the ionic currents mentioned above, I_{L} and I_{inj} are the leak and the external input currents, respectively. The leak current is given by Ohm's law:

$$I_{\mathrm{L}} = g_{\mathrm{L}} \cdot (V - V_{\mathrm{L}}) \quad (2)$$

nca-1 and *nca-2* encode the homologue of calcium-permeable NALCN channel, which can be modeled as leak channels [1]:

$$I_{\mathrm{NCA}} = g_{\mathrm{NCA}} \cdot (V - V_{\mathrm{Na}}) \quad (3)$$

EXP-2 is a repolarization-activated HERG (Kv11.1, KCNH2) potassium channel homolog in *C. elegans* [2,3]. Because of its unusual gating characteristics, the EXP-2 gating current cannot be directly modeled within the Hodgkin-Huxley formulation (it has no existing ordinary-differential-equation model yet). Considering the similarity in kinetic properties between EXP-2 and HERG channel [2], we used a Markov chain model of HERG channel based on the kinetic scheme from the work of Mazhari et al. to model the EXP-2 channel [4]. The Markov model can be represented by the following five-state diagram:

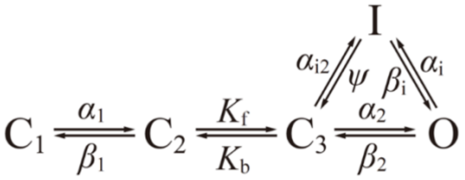

where C_1, C_2 and C_3 are the close states, O is the open state, and I is an inactive state. The forms of the various rate constants are as follows:

$$\alpha_1(V) = p_1 \mathrm{e}^{p_2 V} \quad (4)$$

$$\beta_1(V) = p_3 \mathrm{e}^{-p_4 V} \quad (5)$$

$$K_{\mathrm{f}} = p_5 \quad (6)$$

$$K_{\mathrm{b}} = p_6 \tag{7}$$

$$\alpha_2(V) = p_7 \mathrm{e}^{p_8 V} \tag{8}$$

$$\beta_2(V) = p_9 \mathrm{e}^{-p_{10} V} \tag{9}$$

$$\alpha_{\mathrm{i}}(V) = p_{11} \mathrm{e}^{p_{12} V} \tag{10}$$

$$\beta_{\mathrm{i}}(V) = p_{13} \mathrm{e}^{-p_{14} V} \tag{11}$$

$$\alpha_{\mathrm{i2}}(V) = p_{15} \mathrm{e}^{p_{16} V} \tag{12}$$

$$\psi(V) = \frac{\beta_2(V) \cdot \beta_{\mathrm{i}}(V) \cdot \alpha_{\mathrm{i2}}(V)}{\alpha_2(V) \cdot \alpha_{\mathrm{i}}(V)} \tag{13}$$

To incorporate the Markov model of the EXP-2 kinetics into the Hodgkin-Huxley formulation, we modified the parameters $p_1 \sim p_{16}$ by fitting to the experimental I-V curve of the repolarization-activated current in Fig. 3a. The dynamics of the Markov model can then be described by the following ordinary differential equations (ODE):

$$\frac{\mathrm{d}C_1}{\mathrm{d}t} = \beta_1(V) \cdot C_2 - \alpha_1(V) \cdot C_1 \tag{14}$$

$$\frac{\mathrm{d}C_2}{\mathrm{d}t} = \alpha_1(V) \cdot C_1 + K_{\mathrm{b}} \cdot C_3 - \left[\beta_1(V) + K_{\mathrm{f}}\right] \cdot C_2 \tag{15}$$

$$\frac{\mathrm{d}C_3}{\mathrm{d}t} = K_{\mathrm{f}} \cdot C_2 + \psi(V) \cdot I + \beta_2(V) \cdot O - \left[K_{\mathrm{b}} + \alpha_{\mathrm{i2}}(V) + \alpha_2(V)\right] \cdot C_3 \tag{16}$$

$$\frac{\mathrm{d}O}{\mathrm{d}t} = \beta_{\mathrm{i}}(V) \cdot I + \alpha_2(V) \cdot C_3 - \left[\beta_2(V) + \alpha_{\mathrm{i}}(V)\right] \cdot O \tag{17}$$

$$I = 1 - C_1 - C_2 - C_3 - O \tag{18}$$

With this ODE formulation of the EXP-2 kinetics, the EXP-2 potassium current is given by

$$I_{\mathrm{EXP2}} = g_{\mathrm{EXP2}} \cdot O \cdot (V - V_{\mathrm{K}}) \tag{19}$$

The calcium channel UNC-2, EGL-19, CCA-1 and the potassium channel SHL-1, EGL-36 were modeled based on Hodgkin-Huxley formulation. The UNC-2 (CaV2) calcium current is given by

$$I_{\mathrm{UNC2}} = g_{\mathrm{UNC2}} \cdot m_{\mathrm{UNC2}}^2 \cdot h_{\mathrm{UNC2}} \cdot (V - V_{\mathrm{Ca}}) \tag{20}$$

where g_{UNC2} is the maximum conductance and V_{Ca} is the calcium reversal potential. The dynamics of the activation and inactivation gating variables, m_{UNC2} and h_{UNC2}. are described by

$$\frac{\mathrm{d}m_{\mathrm{UNC2}}}{\mathrm{d}t} = \alpha_{m_{\mathrm{UNC2}}}(V)(1-m_{\mathrm{UNC2}}) - \beta_{m_{\mathrm{UNC2}}}(V)m_{\mathrm{UNC2}} \tag{21}$$

$$\frac{\mathrm{d}h_{\mathrm{UNC2}}}{\mathrm{d}t} = \alpha_{h_{\mathrm{UNC2}}}(V)(1-h_{\mathrm{UNC2}}) - \beta_{h_{\mathrm{UNC2}}}(V)h_{\mathrm{UNC2}} \tag{22}$$

$$\alpha_{m_{\mathrm{UNC2}}}(V) = \frac{a(V-b)}{1-\mathrm{e}^{-\left(\frac{V-b}{c}\right)}} \tag{23}$$

$$\beta_{m_{\mathrm{UNC2}}}(V) = a\mathrm{e}^{-\left(\frac{V-b}{c}\right)} \tag{24}$$

$$\alpha_{h_{\mathrm{UNC2}}}(V) = a\mathrm{e}^{-\left(\frac{V-b}{c}\right)} \tag{25}$$

$$\beta_{h_{\mathrm{UNC2}}}(V) = \frac{a}{1+\mathrm{e}^{-\left(\frac{V-b}{c}\right)}} \tag{26}$$

where $\alpha_{m_{\mathrm{UNC2}}}, \alpha_{h_{\mathrm{UNC2}}}, \beta_{m_{\mathrm{UNC2}}}$ and $\beta_{h_{\mathrm{UNC2}}}$ are the rate constants of m_{UNC2} and h_{UNC2}, and a, b and c are parameters**.**

We follow the work of Nicoletti et al. to model the EGL-19, CCA-1 and SHL-1 channels [1]. The EGL-19 (CaV1) L-type calcium current is given by

$$I_{\mathrm{EGL19}} = g_{\mathrm{EGL19}} \cdot m_{\mathrm{EGL19}} \cdot h_{\mathrm{EGL19}} \cdot (V - V_{\mathrm{Ca}}) \tag{27}$$

where g_{EGL19} is the maximum conductance. The dynamics of the activation and inactivation gating variables, m_{EGL19} and h_{EGL19}, are described by

$$\frac{\mathrm{d}m_{\mathrm{EGL19}}}{\mathrm{d}t} = \frac{m_{\mathrm{EGL19},\infty}(V) - m_{\mathrm{EGL19}}}{\tau_{m_{\mathrm{EGL19}}}(V)} \tag{28}$$

$$\frac{\mathrm{d}h_{\mathrm{EGL19}}}{\mathrm{d}t} = \frac{h_{\mathrm{EGL19},\infty}(V) - h_{\mathrm{EGL19}}}{\tau_{h_{\mathrm{EGL19}}}(V)} \tag{29}$$

$$m_{\mathrm{EGL19},\infty}(V) = \frac{1}{1+\mathrm{e}^{-\left(\frac{V-V_{1/2}}{k_a}\right)}} \tag{30}$$

$$h_{\mathrm{EGL19},\infty}(V)=\left[\frac{a}{1+\mathrm{e}^{-\left(\frac{V-V_{1/2,1}}{k_{i,1}}\right)}}+b\right]\cdot\left[\frac{c}{1+\mathrm{e}^{\left(\frac{V-V_{1/2,2}}{k_{i,2}}\right)}}+d\right] \tag{31}$$

$$\tau_{m_{\mathrm{EGL19}}}(V)=\left[a\mathrm{e}^{-\left(\frac{V-b}{c}\right)^2}\right]+\left[d\mathrm{e}^{-\left(\frac{V-e}{f}\right)^2}\right]+g \tag{32}$$

$$\tau_{h_{\mathrm{EGL19}}}(V)=a\left[\frac{b}{1+\mathrm{e}^{\left(\frac{V-c}{d}\right)}}+\frac{e}{1+\mathrm{e}^{\left(\frac{V-f}{g}\right)}}+h\right] \tag{33}$$

where $m_{\mathrm{EGL19},\infty}$ and $h_{\mathrm{EGL19},\infty}$ are the steady-state functions of the gating variables, $\tau_{m_{\mathrm{EGL19}}}$ and $\tau_{h_{\mathrm{EGL19}}}$ are the corresponding voltage-dependent time constants.

The CCA-1 and SHL-1 channels were modeled similarly to EGL-19; their equations are listed below.

CCA-1 (CaV3) T-type calcium current:

$$I_{\mathrm{CCA1}}=g_{\mathrm{CCA1}}\cdot m_{\mathrm{CCA1}}^2\cdot h_{\mathrm{CCA1}}\cdot(V-V_{\mathrm{Ca}}) \tag{34}$$

$$\frac{\mathrm{d}m_{\mathrm{CCA1}}}{\mathrm{d}t}=\frac{m_{\mathrm{CCA1},\infty}(V)-m_{\mathrm{CCA1}}}{\tau_{m_{\mathrm{CCA1}}}(V)} \tag{35}$$

$$\frac{\mathrm{d}h_{\mathrm{CCA1}}}{\mathrm{d}t}=\frac{h_{\mathrm{CCA1},\infty}(V)-h_{\mathrm{CCA1}}}{\tau_{h_{\mathrm{CCA1}}}(V)} \tag{36}$$

$$m_{\mathrm{CCA1},\infty}(V)=\frac{1}{1+\mathrm{e}^{-\left(\frac{V-V_{1/2}}{k_a}\right)}} \tag{37}$$

$$h_{\mathrm{CCA1},\infty}(V)=\frac{1}{1+\mathrm{e}^{\left(\frac{V-V_{1/2}}{k_i}\right)}} \tag{38}$$

$$\tau_{m_{\mathrm{CCA1}}}(V)=\frac{a}{1+\mathrm{e}^{-\left(\frac{V-b}{c}\right)}}+d \tag{39}$$

$$\tau_{h_{\mathrm{CCA1}}}(V)=\frac{a}{1+\mathrm{e}^{\left(\frac{V-b}{c}\right)}}+d \tag{40}$$

SHL-1 (Kv4) potassium current:

$$I_{\mathrm{SHL1}} = g_{\mathrm{SHL1}} \cdot m_{\mathrm{SHL1}}^{3} \cdot (0.7h_{\mathrm{SHL1,f}} + 0.3h_{\mathrm{SHL1,s}}) \cdot (V - V_{\mathrm{K}}) \quad (41)$$

$$\frac{\mathrm{d}m_{\mathrm{SHL1}}}{\mathrm{d}t} = \frac{m_{\mathrm{SHL1},\infty}(V) - m_{\mathrm{SHL1}}}{\tau_{m_{\mathrm{SHL1}}}(V)} \quad (42)$$

$$\frac{\mathrm{d}h_{\mathrm{SHL1,f}}}{\mathrm{d}t} = \frac{h_{\mathrm{SHL1},\infty}(V) - h_{\mathrm{SHL1,f}}}{\tau_{h_{\mathrm{SHL1,f}}}(V)} \quad (43)$$

$$\frac{\mathrm{d}h_{\mathrm{SHL1,s}}}{\mathrm{d}t} = \frac{h_{\mathrm{SHL1},\infty}(V) - h_{\mathrm{SHL1,s}}}{\tau_{h_{\mathrm{SHL1,s}}}(V)} \quad (44)$$

$$m_{\mathrm{SHL1},\infty}(V) = \frac{1}{1 + \mathrm{e}^{-\left(\frac{V - V_{1/2}}{k_a}\right)}} \quad (45)$$

$$h_{\mathrm{SHL1},\infty}(V) = \frac{1}{1 + \mathrm{e}^{\left(\frac{V - V_{1/2}}{k_i}\right)}} \quad (46)$$

$$\tau_{m_{\mathrm{SHL1}}}(V) = \frac{a}{\mathrm{e}^{-\left(\frac{V-b}{c}\right)} + \mathrm{e}^{\left(\frac{V-d}{e}\right)}} + f \quad (47)$$

$$\tau_{h_{\mathrm{SHL1,f}}}(V) = \frac{a}{1 + \mathrm{e}^{\left(\frac{V-b}{c}\right)}} + d \quad (48)$$

$$\tau_{h_{\mathrm{SHL1,s}}}(V) = \frac{a}{1 + \mathrm{e}^{\left(\frac{V-b}{c}\right)}} + d \quad (49)$$

The action potential in the *exp-2;shl-1* double mutants (Fig. S2d) indicated that AVL (and hence the model) required additional potassium channels that open at depolarized membrane potentials to provide outward current. We noted that *egl-36* encodes a *Shaw* subfamily (Kv3) of voltage-gated potassium channels which is expressed in AVL [5,6]. Here we added EGL-36 into the model and with parameters based on experimental data of Johnstone et al. (1997). The EGL-36 (Kv3) potassium current activates with three components at fast, medium and slow timescales, and it is modeled by the equations below

$$I_{\mathrm{EGL36}} = g_{\mathrm{EGL36}} \cdot (0.33m_{\mathrm{EGL36,f}} + 0.36m_{\mathrm{EGL36,m}} + 0.39m_{\mathrm{EGL36,s}}) \cdot (V - V_{\mathrm{K}}) \quad (50)$$

$$\frac{\mathrm{d}m_{\mathrm{EGL36,f}}}{\mathrm{d}t} = \frac{m_{\mathrm{EGL36},\infty}(V) - m_{\mathrm{EGL36,f}}}{\tau_{m_{\mathrm{EGL36,f}}}} \quad (51)$$

$$\frac{\mathrm{d}m_{\mathrm{EGL36,m}}}{\mathrm{d}t} = \frac{m_{\mathrm{EGL36},\infty}(V) - m_{\mathrm{EGL36,m}}}{\tau_{m_{\mathrm{EGL36,m}}}} \quad (52)$$

$$\frac{\mathrm{d}m_{\mathrm{EGL36,s}}}{\mathrm{d}t} = \frac{m_{\mathrm{EGL36},\infty}(V) - m_{\mathrm{EGL36,s}}}{\tau_{m_{\mathrm{EGL36,s}}}} \tag{53}$$

$$m_{\mathrm{EGL36},\infty}(V) = \frac{1}{1 + \mathrm{e}^{-\left(\frac{V - V_{1/2}}{k_a}\right)}} \tag{54}$$

$$\tau_{m_{\mathrm{EGL36,f}}} = \tau_{\mathrm{f}} \tag{55}$$

$$\tau_{m_{\mathrm{EGL36,m}}} = \tau_{\mathrm{m}} \tag{56}$$

$$\tau_{m_{\mathrm{EGL36,s}}} = \tau_{\mathrm{s}} \tag{57}$$

During our modeling of individual ionic currents, the conductance of each channel was estimated to fit the current and voltage clamp experiment. We attempted to predict the phenotypes of four potassium channel mutants *(exp-2(gf), exp-2(lf), shl-1* and *exp-2;shl-1* double mutant) by modifying the parameters and fitting it to the experimental mutant data. For the *exp-2(gf)* mutant, the parameters of EXP-2 Markov model was modified by fitting to the current-voltage curve of the repolarization-activated current of *exp-2(gf)* in Fig. 3b. For the loss-of-function mutants, the specific mutant channel was knocked out *in silico* by setting its conductance to zero and increasing conductance of other potassium channels.

All our simulations were written in MATLAB and the parameter values of the wildtype and mutant models can be found in Supplementary Table 1.

Supplementary Table 1

AVL Model Parameters

Channel	Function	Parameter	WT	*exp-2(lf)*	*exp-2(gf)*	*shl-1*	*exp-2;shl-1*	Unit
		C	5	5	5	5	5	pF
		V_{K}	-84	-84	-84	-84	-84	mV
		V_{Ca}	100	100	100	100	100	mV
Leak		g_{L}	0.27	0.27	0.27	0.27	0.27	nS
		V_{L}	-60	-60	-60	-60	-60	mV
NCA		g_{NCA}	0.02	0.02	0.02	0.02	0.02	nS
		V_{Na}	30	30	30	30	30	mV
UNC-2		g_{UNC2}	1	1	1	1	1	nS
	$\alpha_{m_{\mathrm{UNC2}}}(V)$	a	0.1	0.1	0.1	0.1	0.1	$ms^{-1} \cdot mV^{-1}$
		b	25	25	25	25	25	mV
		c	10	10	10	10	10	mV
	$\beta_{m_{\mathrm{UNC2}}}(V)$	a	0.4	0.4	0.4	0.4	0.4	ms^{-1}
		b	-25	-25	-25	-25	-25	mV
		c	18	18	18	18	18	mV
	$\alpha_{h_{\mathrm{UNC2}}}(V)$	a	0.01	0.01	0.01	0.01	0.01	ms^{-1}
		b	-50	-50	-50	-50	-50	mV
		c	10	10	10	10	10	mV
	$\beta_{h_{\mathrm{UNC2}}}(V)$	a	0.03	0.03	0.03	0.03	0.03	ms^{-1}
		b	-17	-17	-17	-17	-17	mV
		c	17	17	17	17	17	mV
EGL-19		g_{EGL19}	0.75	0.75	0.75	0.75	0.75	nS
	$\tau_{m_{\mathrm{EGL19}}}(V)$	a	2.9	2.9	2.9	2.9	2.9	ms
		b	-4.8	-4.8	-4.8	-4.8	-4.8	mV
		c	6	6	6	6	6	mV
		d	1.9	1.9	1.9	1.9	1.9	ms
		e	-8.6	-8.6	-8.6	-8.6	-8.6	mV

		f	30	30	30	30	30	mV
		g	2.3	2.3	2.3	2.3	2.3	ms
	$\tau_{h_{\mathrm{EGL19}}}(V)$	a	0.4	0.4	0.4	0.4	0.4	
		b	44.6	44.6	44.6	44.6	44.6	ms
		c	-33	-33	-33	-33	-33	mV
		d	5	5	5	5	5	mV
		e	36.4	36.4	36.4	36.4	36.4	ms
		f	18.7	18.7	18.7	18.7	18.7	mV
		g	3.7	3.7	3.7	3.7	3.7	mV
		h	43.1	43.1	43.1	43.1	43.1	ms
	$m_{\mathrm{EGL19},\infty}(V)$	$V_{1/2}$	-4.4	-4.4	-4.4	-4.4	-4.4	mV
		k_a	7.5	7.5	7.5	7.5	7.5	mV
	$h_{\mathrm{EGL19},\infty}(V)$	a	1.43	1.43	1.43	1.43	1.43	
		$V_{1/2,1}$	14.9	14.9	14.9	14.9	14.9	mV
		$k_{i,1}$	12	12	12	12	12	mV
		b	0.14	0.14	0.14	0.14	0.14	
		c	5.96	5.96	5.96	5.96	5.96	
		$V_{1/2,2}$	-20.5	-20.5	-20.5	-20.5	-20.5	mV
		$k_{i,2}$	8.1	8.1	8.1	8.1	8.1	mV
		d	0.6	0.6	0.6	0.6	0.6	
CCA-1		g_{CCA1}	0.25	0.25	0.25	0.25	0.25	nS
	$m_{\mathrm{CCA1},\infty}(V)$	$V_{1/2}$	-43.32	-43.32	-43.32	-43.32	-43.32	mV
		k_a	7.6	7.6	7.6	7.6	7.6	mV
	$\tau_{m_{\mathrm{CCA1}}}(V)$	a	40	40	40	40	40	ms
		b	-62.5	-62.5	-62.5	-62.5	-62.5	mV
		c	-12.6	-12.6	-12.6	-12.6	-12.6	mV
		d	0.7	0.7	0.7	0.7	0.7	ms
	$h_{\mathrm{CCA1},\infty}(V)$	$V_{1/2}$	-58	-58	-58	-58	-58	mV

		k_i	7	7	7	7	7	mV
	$\tau_{h_{\mathrm{CCA1}}}(V)$	a	280	280	280	280	280	ms
		b	-60.7	-60.7	-60.7	-60.7	-60.7	mV
		c	8.5	8.5	8.5	8.5	8.5	mV
		d	19.8	19.8	19.8	19.8	19.8	ms
SHL1		g_{SHL1}	4	5.4	4	0	0	nS
	$m_{\mathrm{SHL1},\infty}(V)$	$V_{1/2}$	-6.8	-6.8	-6.8	-6.8	-6.8	mV
		k_a	14.1	14.1	14.1	14.1	14.1	mV
	$\tau_{m_{\mathrm{SHL1}}}(V)$	a	1.4	1.4	1.4	1.4	1.4	ms
		b	-17.5	-17.5	-17.5	-17.5	-17.5	mV
		c	12.9	12.9	12.9	12.9	12.9	mV
		d	-3.7	-3.7	-3.7	-3.7	-3.7	mV
		e	6.5	6.5	6.5	6.5	6.5	mV
		f	0.2	0.2	0.2	0.2	0.2	ms
	$h_{\mathrm{SHL1},\infty}(V)$	$V_{1/2}$	-33.1	-33.1	-33.1	-33.1	-33.1	mV
		k_i	8.3	8.3	8.3	8.3	8.3	mV
	$\tau_{h_{\mathrm{SHL1,f}}}(V)$	a	5.9	5.9	5.9	5.9	5.9	ms
		b	-8.2	-8.2	-8.2	-8.2	-8.2	mV
		c	2.9	2.9	2.9	2.9	2.9	mV
		d	2.73	2.73	2.73	2.73	2.73	ms
	$\tau_{h_{\mathrm{SHL1,s}}}(V)$	a	84.2	84.2	84.2	84.2	84.2	ms
		b	-7.7	-7.7	-7.7	-7.7	-7.7	mV
		c	2.4	2.4	2.4	2.4	2.4	mV
		d	11.9	11.9	11.9	11.9	11.9	ms
EGL-36		g_{EGL36}	1.35	1.8225	1.35	2.025	2.7	nS
	$m_{\mathrm{EGL36},\infty}(V)$	$V_{1/2}$	63	63	63	63	63	mV
		k_a	28.5	28.5	28.5	28.5	28.5	mV
	$\tau_{m_{\mathrm{EGL36,f}}}$	τ_{f}	13	13	13	13	13	ms

	$\tau_{m_{\mathrm{EGL36,m}}}$	τ_{m}	63	63	63	63	63	ms
	$\tau_{m_{\mathrm{EGL36,s}}}$	τ_{s}	355	355	355	355	355	ms
EXP-2		g_{EXP2}	3.1	0	3.1	4.65	0	nS
	$\alpha_1(V)$	p_1	0.0241	0.0241	0.0097	0.0241	0.0241	ms^{-1}
		p_2	0.0408	0.0408	0.0367	0.0408	0.0408	mV^{-1}
	$\beta_1(V)$	p_3	0.0091	0.0091	0.002	0.0091	0.0091	ms^{-1}
		p_4	0.03	0.03	0.00082	0.03	0.03	mV^{-1}
	K_{f}	p_5	0.0372	0.0372	0.0479	0.0372	0.0372	ms^{-1}
	K_{b}	p_6	0.31	0.31	0.31	0.31	0.31	ms^{-1}
	$\alpha_2(V)$	p_7	0.0376	0.0376	0.0811	0.0376	0.0376	ms^{-1}
		p_8	0.0472	0.0472	0.0367	0.0472	0.0472	mV^{-1}
	$\beta_2(V)$	p_9	0.0015	0.0015	0.0012	0.0015	0.0015	ms^{-1}
		p_{10}	0.0703	0.0703	0.0511	0.0703	0.0703	mV^{-1}
	$\alpha_{\mathrm{i}}(V)$	p_{11}	0.2177	0.2177	0.1182	0.2177	0.2177	ms^{-1}
		p_{12}	0.03	0.03	0.006	0.03	0.03	mV^{-1}
	$\beta_{\mathrm{i}}(V)$	p_{13}	0.0313	0.0313	0.0313	0.0313	0.0313	ms^{-1}
		p_{14}	0.1418	0.1418	0.1418	0.1418	0.1418	mV^{-1}
	$\alpha_{\mathrm{i2}}(V)$	p_{15}	8.72E-06	8.72E-06	8.7204E-06	8.72E-06	8.72E-06	ms^{-1}
		p_{16}	1.40E-06	1.40E-06	1.4011E-06	1.40E-06	1.40E-06	mV^{-1}

Supplementary Reference

1 Nicoletti, M. *et al.* Biophysical modeling of *C. elegans* neurons: Single ion currents and whole-cell dynamics of AWCon and RMD. *PloS one* **14**, e0218738, doi:10.1371/journal.pone.0218738 (2019).

2 Fleischhauer, R. *et al.* Ultrafast inactivation causes inward rectification in a voltage-gated K(+) channel from *Caenorhabditis elegans*. *The Journal of neuroscience : the official journal of the Society for Neuroscience* **20**, 511-520 (2000).

3 Shtonda, B. & Avery, L. CCA-1, EGL-19 and EXP-2 currents shape action potentials in the *Caenorhabditis elegans* pharynx. *J Exp Biol* **208**, 2177-2190, doi:10.1242/jeb.01615 (2005).

4 Mazhari, R., Greenstein, J. L., Winslow, R. L., Marbán, E. & Nuss, H. B. Molecular interactions between two long-QT syndrome gene products, HERG and KCNE2, rationalized by in vitro and in silico analysis. *Circ Res* **89**, 33-38, doi:10.1161/hh1301.093633 (2001).

5 Johnstone, D. B., Wei, A., Butler, A., Salkoff, L. & Thomas, J. H. Behavioral defects in *C. elegans* egl-36 mutants result from potassium channels shifted in voltage-dependence of activation. *Neuron* **19**, 151-164, doi:10.1016/s0896-6273(00)80355-4 (1997).

6 Choi, U., Wang, H., Hu, M., Kim, S. & Sieburth, D. Presynaptic coupling by electrical synapses coordinates a rhythmic behavior by synchronizing the activities of a neuron pair. *Proceedings of the National Academy of Sciences of the United States of America* **118**, doi:10.1073/pnas.2022599118 (2021).
