# Supplementary material for: C. elegans enteric motor neurons fire synchronized action potentials underlying the defecation motor program
Source: Nat Commun. 2022 May 19;13:2783. doi: 10.1038/s41467-022-30452-y (PMC9120479; doi:10.1038/s41467-022-30452-y)
Supplement: Supplementary file 3 — Description to Additional Supplementary Information [file 41467_2022_30452_MOESM3_ESM.pdf]

Description of Additional Supplementary Files

Supplementary Movie 1

The movie used for Figure 5 in real time (the beginning of the movie was slightly chopped). GCaMP6f was expressed in intestine, AVL, DVB, DVC and non-specifically in pharynx. At ~2 sec of the movie the gut calcium wave was initiated at the anterior gut and quickly spread to the whole intestine. At ~5 sec, AVL and DVB cell body as well as the axon between the two neurons across the whole-body length fired near-synchronized calcium spikes.

Supplementary Movie 2

A real-time movie showing a defecation cycle with dual-color florescence. GCaMP6f and mCherry were co-expressed in the intestine and all GABA neurons. At ~3 sec of the movie the gut calcium wave was initiated at the anterior gut and quickly spread to the whole intestine. At ~11sec, AVL and DVB cell body as well as the axon between the two neurons across the whole-body length fired near-synchronized calcium spikes. Calcium spikes in the axon in both the head and the tail can be seen slightly preceding the cell-body spikes. mCherry florescence was not changed throughout the movie.
