# Supplementary material for: C. elegans enteric motor neurons fire synchronized action potentials underlying the defecation motor program
Source: Nat Commun. 2022 May 19;13:2783. doi: 10.1038/s41467-022-30452-y (PMC9120479; doi:10.1038/s41467-022-30452-y)
Supplement: Supplementary file 6 — Reporting Summary [file 41467_2022_30452_MOESM6_ESM.pdf]

nature portfolio

Corresponding author(s): Qiang Liu

Last updated by author(s): Apr 19, 2022

Reporting Summary

Nature Portfolio wishes to improve the reproducibility of the work that we publish. This form provides structure for consistency and transparency in reporting. For further information on Nature Portfolio policies, see our Editorial Policies and the Editorial Policy Checklist.

Statistics

For all statistical analyses, confirm that the following items are present in the figure legend, table legend, main text, or Methods section.

n/a	Confirmed	
☐	☒	The exact sample size (*n*) for each experimental group/condition, given as a discrete number and unit of measurement
☒	☐	A statement on whether measurements were taken from distinct samples or whether the same sample was measured repeatedly
☐	☒	The statistical test(s) used AND whether they are one- or two-sided<br>*Only common tests should be described solely by name; describe more complex techniques in the Methods section.*
☒	☐	A description of all covariates tested
☒	☐	A description of any assumptions or corrections, such as tests of normality and adjustment for multiple comparisons
☐	☒	A full description of the statistical parameters including central tendency (e.g. means) or other basic estimates (e.g. regression coefficient) AND variation (e.g. standard deviation) or associated estimates of uncertainty (e.g. confidence intervals)
☐	☒	For null hypothesis testing, the test statistic (e.g. *F*, *t*, *r*) with confidence intervals, effect sizes, degrees of freedom and *P* value noted<br>*Give P values as exact values whenever suitable.*
☒	☐	For Bayesian analysis, information on the choice of priors and Markov chain Monte Carlo settings
☒	☐	For hierarchical and complex designs, identification of the appropriate level for tests and full reporting of outcomes
☒	☐	Estimates of effect sizes (e.g. Cohen's *d*, Pearson's *r*), indicating how they were calculated

Our web collection on statistics for biologists contains articles on many of the points above.

Software and code

Policy information about availability of computer code

Data collection	Electrophysiology: Patchmaster v2x90 (Heka)<br>Calcium imaging: MATLAB R2018a (MathWorks) Custom code was submitted with this manuscript
Data analysis	Electrophysiology: OriginPro2020 (OriginLab)<br>Calcium imaging and Modeling: MATLAB R2018a (MathWorks) Custom code was submitted with this manuscript

For manuscripts utilizing custom algorithms or software that are central to the research but not yet described in published literature, software must be made available to editors and reviewers. We strongly encourage code deposition in a community repository (e.g. GitHub). See the Nature Portfolio guidelines for submitting code & software for further information.

Data

Policy information about availability of data

All manuscripts must include a data availability statement. This statement should provide the following information, where applicable:

- Accession codes, unique identifiers, or web links for publicly available datasets
- A description of any restrictions on data availability
- For clinical datasets or third party data, please ensure that the statement adheres to our policy

Data used in this manuscript are deposited in the Mendeley Database (doi: 10.17632/33mnszcbh8.1)

Field-specific reporting

Please select the one below that is the best fit for your research. If you are not sure, read the appropriate sections before making your selection.

☒ Life sciences ☐ Behavioural & social sciences ☐ Ecological, evolutionary & environmental sciences

For a reference copy of the document with all sections, see nature.com/documents/nr-reporting-summary-flat.pdf

Life sciences study design

All studies must disclose on these points even when the disclosure is negative.

Sample size	No sample-size calculation was performed. Sample sizes were chosen based on empirical knowledge of sample variability. My previous work published in Cell (Liu et al., 2018) and similar studies published in Nature Communications (Chen et al., 2017; Liu et al., 2017; Liu et al., 2013; 2014; 2020).<br><br>Reference:<br>Chen, B., Liu, P., Hujber, E.J., Li, Y., Jorgensen, E.M., and Wang, Z.W. (2017). AIP limits neurotransmitter release by inhibiting calcium bursts from the ryanodine receptor. Nat Commun 8, 1380. 10.1038/s41467-017-01704-z.<br>Liu, P., Chen, B., Mailler, R., and Wang, Z.W. (2017). Antidromic-rectifying gap junctions amplify chemical transmission at functionally mixed electrical-chemical synapses. Nat Commun 8, 14818. 10.1038/ncomms14818.<br>Liu, P., Chen, B., and Wang, Z.W. (2013). Postsynaptic current bursts instruct action potential firing at a graded synapse. Nat Commun 4, 1911. 10.1038/ncomms2925.<br>Liu, P., Chen, B., and Wang, Z.W. (2014). SLO-2 potassium channel is an important regulator of neurotransmitter release in Caenorhabditis elegans. Nat Commun 5, 5155. 10.1038/ncomms6155.<br>Liu, P., Chen, B., and Wang, Z.W. (2020). GABAergic motor neurons bias locomotor decision-making in C. elegans. Nat Commun 11, 5076. 10.1038/s41467-020-18893-9.<br>Liu, Q., Kidd, P.B., Dobosiewicz, M., and Bargmann, C.I. (2018). C. elegans AWA Olfactory Neurons Fire Calcium-Mediated All-or-None Action Potentials. Cell 175, 57-70 e17. 10.1016/j.cell.2018.08.018.
Data exclusions	Pre-established data exclusion criteria for electrophysiological recording: only patch clamps with a seal resistance above 1 GΩ and uncompensated series resistance below 100 MΩ will be accepted for further analysis.
Replication	Replications were conducted for 1-5 times for any given recordings while the preparations lasts.
Randomization	Animals for experiments were randomly selected from a culture dish with hundreds of animals with the same genotype.
Blinding	No

Reporting for specific materials, systems and methods

We require information from authors about some types of materials, experimental systems and methods used in many studies. Here, indicate whether each material, system or method listed is relevant to your study. If you are not sure if a list item applies to your research, read the appropriate section before selecting a response.

Materials & experimental systems

n/a	Involved in the study	
☒	☐	Antibodies
☒	☐	Eukaryotic cell lines
☒	☐	Palaeontology and archaeology
☐	☒	Animals and other organisms
☒	☐	Human research participants
☒	☐	Clinical data
☒	☐	Dual use research of concern

Methods

n/a	Involved in the study	
☒	☐	ChIP-seq
☒	☐	Flow cytometry
☒	☐	MRI-based neuroimaging

Animals and other organisms

Policy information about studies involving animals; ARRIVE guidelines recommended for reporting animal research

Laboratory animals	3-days old C. elegans hermaphrodites. Wild-type is Bristol strain N2 and its variant strain PD1074
Wild animals	No wild animals were used in the study
Field-collected samples	No field collected samples were used in the study

March 2021

Ethics oversight	This study on invertebrate animal C. elegans did not require an ethical approval.

Note that full information on the approval of the study protocol must also be provided in the manuscript.
